# The incidence risk of type 2 diabetes mellitus in female nurses: a nationwide matched cohort study

**DOI:** 10.1186/s12889-016-3113-y

**Published:** 2016-05-26

**Authors:** Hsiu-Ling Huang, Cheng-Chin Pan, Shun-Mu Wang, Pei-Tseng Kung, Wen-Yu Chou, Wen-Chen Tsai

**Affiliations:** Department of Aged Welfare & Social Work, Toko University, Taiwan, Republic of China; Department of Public Health and Department of Health Services Administration, China Medical University, Taiwan, Republic of China; Department of Urology, Hengchun Tourism Hospital, Ministry of Health and Welfare, Taiwan, Republic of China; Department of Healthcare Administration, Asia University, Taichung, Taiwan Republic of China; Department of Health Services Administration, China Medical University, 91, Hsueh-Shih Road, Taichung, Taiwan Republic of China

**Keywords:** Diabetes, Nurse, Occupational health, National health insurance, Cohort study

## Abstract

**Background:**

Diabetes is one of the most common chronic illnesses worldwide. This study was to assess whether the incidence risk of type 2 diabetes mellitus between female nurses and female non-nurses.

**Methods:**

Study data were obtained from the Longitudinal Health Insurance Research Database, and nurses were sampled from the Registry for medical personnel. Nurses and non-nurses with similar traits and health conditions were selected via 1:1 propensity score matching. A total of 111,670 subjects were selected (55,835 nurses and 55,835 non-nurses). Stages of diabetes development were monitored until December 31, 2009. The Cox proportional hazards model was used to discuss risks and influencing factors related to diabetes. Poisson distribution methods were used to examine the incidence rate of diabetes per 1,000 person-years.

**Results:**

The propensity matching results show that on average, female nurses who were diagnosed with diabetes were younger compared with the non-nurses (46.98 ± 10.80 vs. 48.31 ± 10.43, *p* <0.05). However, the results of the Cox proportional hazards model show that the nurses showed a lower risk of developing diabetes compared with the non-nurses (Adj. HR = 0.84, 95 % CI: 0.79–0.90). Factors influencing diabetes development risks among the nurses include advanced age and high Charlson Comorbidity Index levels.

**Conclusion:**

The low degree of diabetes development among the nurses may be attributable to the fact that nurses possess substantial knowledge on health care and on healthy behaviors. The results of this study can be used as a reference to assess occupational risks facing nursing staff, to prevent diabetes development, and to promote health education.

## Background

Nurses work on the front lines in the medical industry and account for 45.2 % of all professional medical personnel in Taiwan [1]. Numerous medical orders and procedures rely on the work of nurses; therefore, nursing care is highly correlated with care quality levels and with patient treatment outcomes, and nurses play a crucial role in the medical industry.

Ongoing care services are essential to the medical industry. Shift work creates certain occupational problems for nurses [2, 3]. Relevant studies have shown that in addition to causing an increasing number of accidents [4] and higher rates of disease and cancer development [5], engagement in shift work increases one’s likelihood of developing metabolic diseases [6, 7]. Previous studies have revealed that individuals working in occupations that involve shift work show higher risks of developing diabetes compared with those who do not work shifts [8–10].

Diabetes is one of the most common chronic illnesses worldwide. Statistics [11] show that approximately 366 million people worldwide were diagnosed with diabetes in 2011. This number is estimated to increase to 552 million by 2030, accounting for 4.4 % of the global population. In the United States, research has been conducted on the health status of 67,420 nurses from 1976 to 1996, and the results show that 7,401 (11 %) of the nurses were diagnosed with type 2 diabetes mellitus (DM) [12]. Since 2003, diabetes has ranked third among the top 10 causes of death among women in Taiwan [13]. Nurses are exposed to an occupational environment that may be detrimental to their health, which may affect the quality of medical care. The present study compares type 2 DM development risk levels among female nurses to those of the general female citizen population to further identify influencing factors. These data may serve as a foundation for future discussions on occupational health in relation to health promotion, prevention and protection.

## Methods

### Study design

A retrospective and longitudinal method was adopted in this study, and data were sourced from the National Health Insurance Research Database. To comply with privacy measures, personal information was removed from the collected data.

The Taiwanese government created a compulsory National Health Insurance (NHI) system in 1995, a governmental insurance system that all citizens are mandated to be insured under. Since the end of 2012, the NHI coverage rate reached to as high as 99.85 % [14]. The NHI covers costs for outpatient procedures; emergency procedures; and inpatient prescriptions, treatments, and examinations. The NHI database details all medical claims records for the insured, including treatments for chronic illnesses such as diabetes [15, 16].

### Data sources and study participants

Data were sourced from the Longitudinal Health Insurance Research Database of 2000 published by the NHI, which included one million population randomly selected to be representative of whole population in Taiwan and a sample of nurses was selected from the nationwide Registry for Medical Personnel published by the NHI. We used female nurses and female non-nurses as the study participants. Nurses were defined as the nurses were listed in the Registry for Medical Personnel before December 31, 2000. Non-nurses were defined as the participants who had not registered as a licensed medical professional, such as physicians, dentists, physical therapists, nutritionists, and so on before the end of study observation. Participants younger than 20 years and older than 90 years and those diagnosed with type 2 DM prior to December 31, 2000 were excluded. Male nurses were excluded from the study, as most nurses in Taiwan are female (98.92 %) [17]. A total of 374,173 participants were selected, 70,675 of whom were nurses and 303,498 of whom were female non-nurses.

To objectively compare diabetes development risks among female nurses and female non-nurses, 1:1 propensity score matching was used to control for selection bias, and participants with similar characteristics and health conditions were selected from the two groups. After propensity score matching, we assigned 55,835 nurses to the observation group and 55,835 non-nurses to the control group (Table 1). Patterns of diabetes development were monitored until December 31, 2009, covering an average of 9.68 ± 1.06 years (9.72 ± 0.92 and 9.64 ± 1.19 y for nurses and non-nurses, respectively).

**Table 1:** Participants demographics before and after propensity score (PS) matching

Variables		Before PS Matching							After PS Matching (1:1)						
		Total		Non-nurses		Nurse		*p* -value	Total		Non-nurses		Nurse		*p* -value
		*N*	%	*N*	%	*N*	%		*N*	%	*N*	%	*N*	%	
Total participants		374173	100.00	303498	81.11	70675	18.89		111670	100.00	55835	50.00	55835	50.00	
Age								<0.001							1.000
	<25	59213	15.83	39267	12.94	19946	28.22		24912	22.31	12456	22.31	12456	22.31	
	25–34	107621	28.76	76758	25.29	30863	43.67		47271	42.33	23628	42.32	23643	42.34	
	35–44	88584	23.67	74351	24.50	14233	20.14		28221	25.27	14118	25.29	14103	25.26	
	45–54	54406	14.54	49631	16.35	4775	6.76		9550	8.55	4775	8.55	4775	8.55	
	55–64	32444	8.67	31771	10.47	673	0.95		1346	1.21	673	1.21	673	1.21	
	≧65	31905	8.53	31720	10.45	185	0.26		370	0.33	185	0.33	185	0.33	
Average age (Mean, Std)		40.13	15.05	42.20	15.48	31.23	8.48		32.79	8.97	33.04	9.10	32.54	8.83	
Monthly salary(NT$)								<0.001							1.000
	Low-income household	1326	0.35	1312	0.43	14	0.02		28	0.03	14	0.03	14	0.03	
	≦17280	47252	12.63	42573	14.03	4679	6.62		9358	8.38	4679	8.38	4679	8.38	
	17281 ~ 22800	141273	37.76	134297	44.25	6976	9.87		13952	12.49	6976	12.49	6976	12.49	
	22801 ~ 28800	71049	18.99	61248	20.18	9801	13.87		19602	17.55	9801	17.55	9801	17.55	
	28801 ~ 36300	34972	9.35	23113	7.62	11859	16.78		20061	17.96	10023	17.95	10038	17.98	
	36301 ~ 45800	44721	11.95	20443	6.74	24278	34.35		27366	24.51	13680	24.50	13686	24.51	
	45801 ~ 57800	24273	6.49	14628	4.82	9645	13.65		16136	14.45	8068	14.45	8068	14.45	
	≧57801	9291	2.48	5868	1.93	3423	4.84		5167	4.63	2594	4.65	2573	4.61	
	Missing data	16		16											
CCI								<0.001							0.980
	0	93747	25.05	76960	25.36	16787	23.75		28218	25.27	14109	25.27	14109	25.27	
	1 ~ 3	127818	34.16	98810	32.56	29008	41.04		43511	38.96	21796	39.04	21715	38.89	
	4 ~ 6	87823	23.47	71246	23.47	16577	23.46		26529	23.76	13224	23.68	13305	23.83	
	7 ~ 9	42956	11.48	36932	12.17	6024	8.52		9815	8.79	4912	8.80	4903	8.78	
	≧10	21829	5.83	19550	6.44	2279	3.22		3597	3.22	1794	3.21	1803	3.23	
Average CCI(Mean, Std)		3.38	3.34	3.48	3.43	2.93	2.89		2.93	2.90	2.92	2.89	2.93	2.92	

CCI, Charlson Comorbidity Index; PS, propensity score

It’s 30 New Taiwan Dollar (NT$) per US dollar

*p* -value was considered significant at *p* < 0.05

### Variables description

In this study, the type 2 diabetes mellitus was defined as a primary or secondary diagnosis with ICD-9-CM: 250 or A-code: A181 and the patients had made 3 or more clinic visits or been hospitalized at least once within 365 days [18], and this study excluded other types DM patients. We categorized residence areas into 7 levels, and level 1 was the most urbanized [19]. We used the modified Charlson Comorbidity Index (CCI) to classify the severity of comorbidity [20]. The CCI involved 17 comorbidities weighted based on severity. Higher score denoted greater comorbidity. Presence of catastrophic illnesses was defined as yes or no. The catastrophic illnesses or injuries were defined by National Health Insurance Administration in Taiwan including 30 categories of major illnesses (e.g., cancer, stroke, hemophilia, type I diabetes, autoimmune diseases, end-stage renal disease etc.) for which patients were exempted from copayment and thus avoided financial hardship [21].

### Data analysis

A descriptive analysis was conducted to examine demographical traits of the research population (e.g., age, monthly salary, and CCI) and to classify the participants as nurses and non-nurses. Propensity matching (PS) methods were used, and a Chi-square test was subsequently employed to compare variations in diabetes development risks for the two groups. The Cox proportional hazards model was used to discuss relative risks and influencing factors related to diabetes. Hazard ratios (HRs) and 95 % CIs were derived from Cox proportional hazards models. Poisson distribution methods were employed to examine diabetes incidence rates for 1,000 person-years for the two groups. In this study, statistic significance was set at *p* < 0.05.

## Results

### Participants demographics

The data prior to propensity matching revealed significant differences (*p* <0.05) between age, monthly salary, and CCI levels for the two groups (Table 1). Before propensity score matching, the average age of the nurses was 31.23 ± 8.48 years, whereas that of the non-nurses was younger by 10.97 ± 7.00 years at 42.20 ± 15.48 years. Most of the nurses (34.35 %) earned a salary of NT$ 36,301–NT$ 45,800, and the non-nurses earned NT$ 17,281–NT$ 22,800 (44.25 %) on average. The CCI scores for the nurses and non-nurses were 2.93 ± 2.89 and 3.48 ± 3.43, respectively.

After performing the propensity score matching tasks, we did not find any significant differences in terms of age, monthly salaries, residence area urbanization levels, CCI levels, and in other catastrophic illness levels between the two groups (Table 1).

### Stratified analysis on the risk of diabetes for the nurses and non-nurses

In the Table 2, the results of the bivariate analysis revealed a lower incidence of diabetes among the nurses (2.68 %) compared with that of the non-nurses (3.13 %), revealing a statistically significant difference (p <0.05). After controlling for other factors, Cox proportional hazards models were used to identify the risk of diabetes for the nurses and non-nurses (Table 3). The results of the nurses showed a lower risk of developing diabetes compared with the non-nurses (Adj. HR = 0.84, 95 % CI: 0.79–0.90). For age, in the 35–44- and 45–54-year age groups, the nurses exhibited a significantly lower risk of diabetes compared with the non-nurses (Adj. HR: 0.82 and 0.75, respectively). For the income groups of less than NT$ 17,280, NT$ 22,801–NT$ 28,800, and NT$ 36,301–NT$ 45,800, nurses presented significantly lower risks of diabetes development compared with those of the non-nurses (Adj. HR: 0.65, 0.77, 0.86, respectively). Regarding urbanization variables, with the exception of participants residing in Levels 4 and 5 urbanization areas, no significant differences in diabetes development risk were found between the nurses and non-nurses (*p* <0.05). In less urbanized regions, nurses showed lower risks of diabetes development than non-nurses (*p* <0.05).

**Table 2:** Bivariate analysis on the incidence of diabetes

Variables		Total		Without Diabetes		Diabetes		*p* -value
		*N*	%	*N*	%	*N*	%	
Total participants		111670	100.00	108422	97.09	3248	2.91	
Nurses or non-nurses								<0.001
	Non-nurses	55835	50.00	54085	96.87	1750	3.13	
	Nurses	55835	50.00	54337	97.32	1498	2.68	
Age								<0.001
	<25	24912	22.31	24695	99.13	217	0.87	
	25–34	47271	42.33	46626	98.64	645	1.36	
	35–44	28221	25.27	27085	95.97	1136	4.03	
	45–54	9550	8.55	8562	89.65	988	10.35	
	55–64	1346	1.21	1149	85.36	197	14.64	
	≧65	370	0.33	305	82.43	65	17.57	
Average age (Mean, Std)		32.79	8.97	32.52	8.78	41.80	10.61	
Monthly salary(NT$)								<0.001
	≦17280	9386	8.41	9087	96.81	299	3.19	
	17281 ~ 22800	13952	12.49	13558	97.18	394	2.82	
	22801 ~ 28800	19602	17.55	19117	97.53	485	2.47	
	28801 ~ 36300	20061	17.96	19626	97.83	435	2.17	
	36301 ~ 45800	27366	24.51	26466	96.71	900	3.29	
	45801 ~ 57800	16136	14.45	15660	97.05	476	2.95	
	≧57801	5167	4.63	4908	94.99	259	5.01	
Urbanization of residence								0.662
	Level 1	42449	38.01	41210	97.08	1239	2.92	
	Level 2 & 3	52585	47.09	51079	97.14	1506	2.86	
	Level 4 & 5	12212	10.94	11849	97.03	363	2.97	
	Level 6 & 7	4424	3.96	4284	96.84	140	3.16	
Other catastrophic illnesses								<0.001
	No	109183	97.77	106068	97.15	3115	2.85	
	Yes	2487	2.23	2354	94.65	133	5.35	
CCI								<0.001
	0	15926	14.26	15894	99.80	32	0.20	
	1 ~ 3	44302	39.67	43328	97.80	974	2.20	
	4 ~ 6	31181	27.92	30138	96.66	1043	3.34	
	7 ~ 9	14076	12.60	13342	94.79	734	5.21	
	≧10	6185	5.54	5720	92.48	465	7.52	
Average CCI (Mean, Std)		3.75	3.16	3.69	3.13	5.71	3.48	

CCI, Charlson Comorbidity Index; HR, hazard ratio; CI, confidence interval

It’s 30 New Taiwan Dollar (NT$) per US dollar

Urbanization level of residence area (overall 7 levels; Level 1 was the most urbanized)

*p* -value was considered significant at *p* < 0.05

**Table 3:** Stratified Cox proportional hazard model analysis on the risk of diabetes for the nurses and non-nurses

Variables		Non-nurses			Nurses			Adj. HR* (Nurse:GP)	95 % CI		*p* -value
		Total	Diabetes (*N*)	Diabetes (%)	Total	Diabetes (*N*)	Diabetes (%)				
Total		55835	1750	3.13	55835	1498	2.68	0.84	0.79	0.90	<0.001
Age(y/o)											
	<35	36084	434	2.23	36099	428	2.24	0.97	0.85	1.11	0.628
	35–44	14118	617	4.37	14103	519	3.68	0.82	0.73	0.92	<0.001
	45–54	4775	563	11.79	4775	425	8.90	0.75	0.66	0.85	<0.001
	55–64	673	104	15.45	673	93	13.82	0.85	0.64	1.13	0.260
	≧65	185	32	17.30	185	33	17.84	0.98	0.60	1.62	0.943
Monthly salary(NT$)											
	≦17280	4693	176	3.75	4693	123	2.62	0.65	0.52	0.82	<0.001
	17281 ~ 22800	6976	211	3.02	6976	183	2.62	0.84	0.69	1.03	0.089
	22801 ~ 28800	9801	273	2.79	9801	212	2.16	0.77	0.64	0.92	0.004
	28801 ~ 36300	10023	218	2.17	10038	217	2.16	0.97	0.81	1.18	0.779
	36301 ~ 45800	13680	479	3.50	13686	421	3.08	0.86	0.76	0.99	0.030
	45801 ~ 57800	8068	251	3.11	8068	225	2.79	0.93	0.78	1.12	0.433
	≧57801	2594	142	5.47	2573	117	4.55	0.80	0.63	1.03	0.080
Urbanization of residence											
	Level 1	22883	695	3.04	19566	544	2.78	0.86	0.76	0.96	0.006
	Level 2 & 3	25499	804	3.15	27086	702	2.59	0.84	0.76	0.93	<0.001
	Level 4 & 5	5544	181	3.26	6668	182	2.73	0.85	0.69	1.05	0.133
	Level 6 & 7	1909	70	3.67	2515	70	2.78	0.71	0.50	1.00	0.050
Other catastrophic illnesses											
	No	54530	1680	3.08	54653	1435	2.63	0.84	0.78	0.90	<0.001
	Yes	1305	70	5.36	1182	63	5.33	0.93	0.66	1.31	0.667
CCI											
	≦3	30433	552	2.70	29795	454	2.10	0.82	0.73	0.93	0.002
	4 ~ 6	15428	551	3.57	15753	492	3.12	0.87	0.77	0.98	0.026
	7 ~ 9	6912	389	5.63	7164	345	4.82	0.85	0.74	0.99	0.030
	≧10	3062	258	8.43	3123	207	6.63	0.80	0.66	0.96	0.015

*The stratified Cox proportional hazards models have controlled for age, monthly salary, urbanization of residence, other catastrophic illnesses, CCI

CCI, Charlson Comorbidity Index; HR, hazard ratio; CI, confidence interval

It’s 30 New Taiwan Dollar (NT$) per US dollar

*p* -value was considered significant at *p* < 0.05

An analysis on the comorbidity of other catastrophic illnesses indicated that risks of diabetes development facing those nurses who did not have other catastrophic illnesses were significantly lower than those of non-nurses (Adj. HR: 0.84; *p* <0.05). While risks of diabetes development among the nurses with catastrophic illnesses were lower than those for the non-nurses, the results were non-significant (*p* <0.05). The results of the CCI analysis show that the nurses presented significantly lower susceptibility to diabetes development overall compared with the non-nurses (*p* <0.05).

### Relative factors and the incidence of diabetes among nurses

The analysis results presented in Table 4 show that age and CCI serves as critical factors that influence diabetes development risks among nurses. Regarding age variables, a 10-year period was used as an interval to create age groups. The older nurses presented relatively higher risks of diabetes development compared with the nurses of less than 25 years (Adj. HR: 1.56–16.85). Nurses who were older than 65 years of age showed a 16.85-fold higher risk of developing diabetes compared with nurses who were younger than 25 years of age (95 % CI: 11.47–24.75).

**Table 4:** Relative factors and the incidence risk of diabetes among nurses

Variables		Unadj. HR	*P*-value	Adj. HR	95 % CI		*p* -value
Age							
	<25 (reference)						
	25–34	1.57	<0.001	1.56	1.30	1.87	<0.001
	35–44	4.52	<0.001	4.19	3.50	5.01	<0.001
	45–54	11.29	<0.001	9.59	7.97	11.55	<0.001
	55–64	18.10	<0.001	13.85	10.67	17.99	<0.001
	≧65	25.34	<0.001	16.85	11.47	24.75	<0.001
Monthly salary(NT$)							
	≦17280(reference)						
	17281 ~ 22800	1.00	0.991	1.23	0.98	1.55	0.077
	22801 ~ 28800	0.83	0.090	1.03	0.82	1.28	0.830
	28801 ~ 36300	0.76	0.015	1.11	0.89	1.38	0.350
	36301 ~ 45800	0.81	0.035	1.06	0.87	1.29	0.588
	45801 ~ 57800	0.92	0.449	0.98	0.79	1.23	0.884
	≧57801	1.42	0.006	1.08	0.84	1.39	0.537
Urbanization of residence							
	Level 1(reference)						
	Level 2 & 3	0.98	0.672	0.97	0.87	1.08	0.572
	Level 4 & 5	1.05	0.545	0.97	0.83	1.14	0.719
	Level 6 & 7	1.11	0.407	0.91	0.72	1.16	0.441
Other catastrophic illnesses							
	No(reference)						
	Yes	2.18	<0.001	1.24	0.97	1.59	0.090
CCI							
	0 (reference)						
	1 ~ 3	12.75	<0.001	12.25	6.74	22.28	<0.001
	4 ~ 6	21.44	<0.001	18.66	10.27	33.90	<0.001
	7 ~ 9	34.13	<0.001	24.85	13.64	45.30	<0.001
	≧10	48.23	<0.001	28.48	15.51	52.29	<0.001

CCI, Charlson Comorbidity Index; HR, hazard ratio; CI, confidence interval

It’s 30 New Taiwan Dollar (NT$) per US dollar

Urbanization level of residence area (overall 7 levels; Level 1 was the most urbanized)

*p* -value was considered significant at *p* < 0.05

CCI significantly influences diabetes development risks among nurses. The analysis results indicate that those nurses with a higher CCI were at a higher risk of developing diabetes than the reference group (CCI = 0). Those nurses with a CCI of ≧10 presented 28.48-fold higher relative risks of diabetes development than the reference group (95 % CI: 15.51–52.29). The research results shown in Table 5 indicate that diabetes incidence rates per 1,000 person-years for the nurses were lower than those of the non-nurses (2.76 ‰ vs. 3.25 ‰). The analysis results on the various variables reveal that with the exception of the ≦25-year age group, the incidence rate of diabetes per 1,000 person-years for nurses of all of the age groups was lower than that of the non-nurses.

**Table 5:** The incidence rate of diabetes per 1000 person-years

Variables		Non-nurses		Nurses		*p* -value
		Diabetes (*N*)	Incidence rate(%)	Diabetes (*N*)	Incidence rate(%)	
Total participants		1750	3.25	1498	2.76	<0.001
Age						
	<25	104	0.87	113	0.94	0.574
	25–34	330	1.44	315	1.36	0.492
	35–44	617	4.53	519	3.78	0.003
	45–54	563	12.63	425	9.41	<0.001
	55–64	104	17.25	93	14.97	0.322
	≧65	32	21.99	33	20.62	0.794
Average age (Mean, Std)		48.31	10.43	46.98	10.80	<0.001
Monthly salary(NT$)						
	≦17280	176	4.07	123	2.71	<0.001
	17281 ~ 22800	211	3.14	183	2.70	0.139
	22801 ~ 28800	273	2.89	212	2.23	0.005
	28801 ~ 36300	218	2.25	217	2.23	0.924
	36301 ~ 45800	479	3.61	421	3.16	0.045
	45801 ~ 57800	251	3.20	225	2.86	0.221
	≧57801	142	5.68	117	4.68	0.120
Urbanization of residence						
	Level 1	695	3.14	544	2.85	0.094
	Level 2 & 3	804	3.28	702	2.67	<0.001
	Level 4 & 5	181	3.40	182	2.82	0.077
	Level 6 & 7	70	3.81	70	2.87	0.093
Other catastrophic illnesses						
	No	1680	3.19	1435	2.70	<0.001
	Yes	70	5.72	63	5.63	0.928
CCI						
	0	22	0.26	10	0.14	0.107
	1 ~ 3	530	2.52	444	2.01	<0.001
	4 ~ 6	551	3.69	492	3.22	0.028
	7 ~ 9	389	5.89	345	5.01	0.029
	≧10	258	9.06	207	7.01	0.006

CCI, Charlson Comorbidity Index

It’s 30 New Taiwan Dollar (NT$) per US dollar

Urbanization level of residence area (overall 7 levels; Level 1 was the most urbanized)

*p* -value was considered significant at *p* < 0.05

## Discussion

**Fig. 1:**
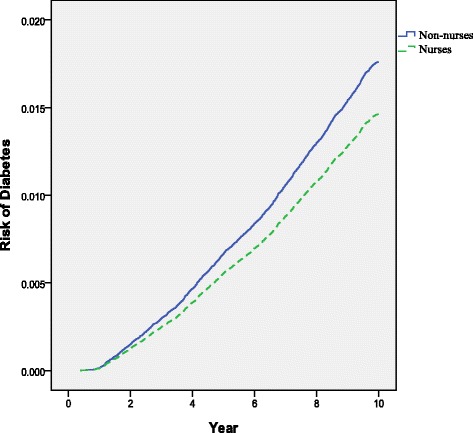
Relative risk of diabetes in nurses and non-nurses

After the research population was collected from the NHI database, 1:1 propensity score matching was employed. The results of the bivariate analysis shown in Table 2 indicate that the nurses presented a lower risk of diabetes development than the non-nurses (2.68 % vs. 3.13 %), which is consistent with that of the Cox proportional hazards model (Table 3 and Fig. 1; Adj. HR: 0.83, 95 % CI: 0.78–0.89).

Previous studies [8–10] have determined that individuals who work in professions that require shift work present higher risks of developing diabetes. After propensity score matching, our nurses cohort and non-nurses control group were similar in terms of demographics, socioeconomic status, and health conditions. However, the nurses showed lower risks of developing diabetes than the non-nurses, and this may be attributed to nurses’ medical knowledge and educational training. Furthermore, nurses are assume health promotion and education responsibilities [22] and are thus likely to live healthy lifestyles and to invest in their long-term health. This phenomenon is known as the healthy worker effect [23, 24]. Nurses must be in excellent health to achieve effective performance; therefore, nurses are generally healthier than non-nurses.

The aforementioned results may be correlated with knowledge, attitudes, and practice (KAP) theory principles [25, 26]. Self-care levels depend on patient behaviors, and a patient’s knowledge influences his or her attitudes, thereby affecting his or her practices. Acquiring correct knowledge about a disease and adopting positive and proactive attitudes are essential for motivating self-care behaviors, which directly or indirectly affect health outcomes. Nurses are equipped with comprehensive medical knowledge and thus possess a favorable view of personal health and diseases and a positive attitude toward medical care, resulting in superior self-care [27]. However, further study is needed to examine the effect of KAP theory on the nurses’ incidence risk of developing diabetes.

The results presented in Tables 3 and 4 show that for the entire sample population, age and CCI variables most heavily influenced diabetes development risks. The older participants presented higher risks of diabetes development than the reference group (<25 y), complementing the results of previous studies [28–30], and this shows that glucose tolerance levels decrease with age; therefore, diabetes development risks increase rapidly among individuals who are older than 45 years of age. As shown in Table 5, a rapid increase in the incidence rate of diabetes per 1,000 person-years was observed among both nurses and non-nurses of 45 years of age and older. As shown in Tables 3 and 4, a higher CCI involves an increased relative risk of diabetes development, complementing the results of Huang [31] and Monami et al. [32]. Thus, CCI serves as a critical variable for predicting diabetes development risks.

### Study limitations

There are several limitations to our analyses. According to the International Statistical Classification of Diseases (ICD), DM diagnosis is based on an ICD-9 diagnosis code. Therefore, clinical diagnostic data could not be acquired and verified. To optimize the accuracy of this study while compensating for this limitation, diabetes occurrence was identified when patients visited an outpatient department three times or when they were hospitalized more than once within 365 days of receiving a primary or secondary diabetes diagnosis (ICD-9-CM: 250 or A-code: A181) [17]. Some risk factors are not present in analysis. For instance, information on lifestyle, health behaviors and clinical testing data were not available and thus could not be used in variable analyses. So, we used the propensity score matching to control for selection bias. The propensity score adjustment is an important statistical technique to reduce the bias from confounding variables in observational studies and mimic the results of a randomized controlled trial [33]. In addition, the long follow-up and national design provided adequate power.

## Conclusion

This study is the first to use a nationwide database to compare risks of diabetes development among female nurses and female non-nurses. The results show that nurses present a lower risk of developing diabetes than non-nurses. This result may be attributable to the fact that nurses possess superior medical knowledge and thus have lower incidence risk of developing diabetes.

## Abbreviations

CCI, Charlson Comorbidity Index; CI, confidence interval; DM, diabetes mellitus; HR, hazard ratio; ICD, International Statistical Classification of Diseases; KAP, knowledge, attitudes, and practice; NHI, National Health Insurance; NT$, New Taiwan Dollar; PS, propensity score.
